# Ultraviolet-B sensitizes silent nociceptors and desensitizes C-touch fibers to slow depolarizing electrical stimuli in pigs

**DOI:** 10.1097/PR9.0000000000001491

**Published:** 2026-08-19

**Authors:** Sabrina Soares, Martin Schmelz, Roman Rukwied

**Affiliations:** Department of Experimental Pain Research, Mannheim Center for Translational Neuroscience, Medical Faculty Mannheim, University of Heidelberg, Heidelberg, Germany

**Keywords:** UV-B inflammation, Slow depolarizing stimulation, Nociceptor sensitization

## Abstract

**Introduction:**

Tetrodotoxin-resistant voltage-sensitive sodium channel subtypes 1.8 and 1.9 play a key role on “silent” (mechano-insensitive) nociceptors activation during sinusoidal electrical stimulation. Therefore, we hypothesized that an UVB–induced sensitization of this particular nociceptor class can be revealed by such electrical stimulation pattern.

**Objectives:**

We set out to study ultraviolet-B (UV-B)-induced sensitization of C-nociceptors using heat and mechanical stimulation, but also transduction independent slow depolarizing electrical pulses.

**Methods:**

Single fibre recordings of pig saphenous nerve were performed after UV-B irradiation with 3-fold minimum erythemal doses.

**Results:**

A total 45 C fibers were recorded from UV-B inflamed skin and discharge patterns compared with nonirradiated control units (n = 134). We did not observe a sensitization to heat or mechanical stimulation in mechanosensitive nociceptors, but some of the “silent” nociceptors were sensitized to mechanical stimulation. The most drastic changes were found for electrical activation thresholds that increased about 3-fold in tactile “C-touch” fibers, but that were reduced in silent nociceptors up to a factor of 5. The threshold to 1-Hz stimulation was also increased 2-fold in “polymodal” nociceptors, although not significantly.

**Conclusion:**

Potentially, the combination of desensitized C-touch and polymodal nociceptors with sensitized silent nociceptors might be based on high expression of depolarization-resistant Na_V_1.8 sodium channels in silent nociceptors. Functionally, sensitized silent nociceptors appear to be the primary candidate to underlie hyperalgesia in acute UV-B inflammation.

## Abstract 2

Supplemental Digital Content is Available in the Text.

Ultraviolet-B–induced inflammation differentially sensitizes single recorded pig C-afferents fibers, particularly desensitizes C-tactile fibers while turning silent nociceptors hyperexcitable.

## 1. Introduction

Ultraviolet-B (UV-B) induced inflammation is associated with local mechanical and thermal hyperalgesia.^2,15,47^ Still, it has been problematic to identify the involved nociceptor class: heat sensitization of polymodal nociceptors was found in the rabbit along with spontaneous C-nociceptor activity, whereas mouse polymodal nociceptors were not sensitized by UV-B.^3^ Moreover, in humans^22^ and pig,^33,48^ UV-B–induced nociceptor sensitization was most evident in mechano-insensitive “silent” nociceptors. Thus, not only species but also test modalities play a role to detect nociceptor class specific UV-B sensitization.

Depolarizing electrical stimulation paradigms have been proven to promote axonal activation preferentially in polymodal and silent C-nociceptors in humans and pigs,^17,34^ an activation which is independent of transduction mechanisms required for mechanical and thermal stimulus signalling. We, therefore, used these stimulation paradigms to identify nociceptor class-specific excitability changes in UV-B–induced inflammation assessing in vivo C-nociceptors in the pig.

Ultraviolet-B is expected to sensitize sensory nerve fiber endings via inflammatory mediators or unspecific factors, such as increased extracellular potassium concentration,^46^ all contributing to a facilitated depolarization. Initially, such depolarisation will increase excitability, but more profound depolarization will also increase inactivation of voltage-sensitive sodium channels, in particular Na_V_1.7, and thereby may reduce excitability.^35^ We hypothesized that nociceptors expressing high activation threshold Na_V_1.8 channels would be predominantly sensitized by UV-B, whereas non-nociceptors lacking Na_V_1.7-Na_V_1.9 might rather be desensitized, thereby providing additional information to our previous report of sensitized mechano-insensitive or “silent” C fibres (CMi) in UV-B–irradiated pig skin.^48^

## 2. Material and methods

### 2.1. Animals

Pigs (*Sus scrofa domesticus*) average weighted 20 to 25 kg and aged 12 ± 4 weeks were sedated with 1 mg/kg of Dormicum (Midazolam, Roche, Switzerland) and 5 mg/kg of Stresnil (Azaperone, Jannsen Pharmaceutica, Belgium). Propofol (Narcofol, cp-pharma, Switzerland) 2 to 4 mg/kg was used to induce general anesthesia, followed by continuous intravenous infusion of 15 to 20 mg/kg/h Narcoren (Pentobarbital-Natrium, Rhone Merieux, Germany). Animals were continuously monitored for EtCO_2_, ECG, body core temperature, SPO_2_, and NIBP (uMEC12 Vet, Mindray, China) and volume-controlled ventilated (E-Vent Plus, Dräger Medical, Germany). Ethical approval was obtained by the regional ethical committee in Karlsruhe, Germany (approval number G-78/18).

### 2.2. Ultraviolet-B–induced inflammation

**Figure 1.**
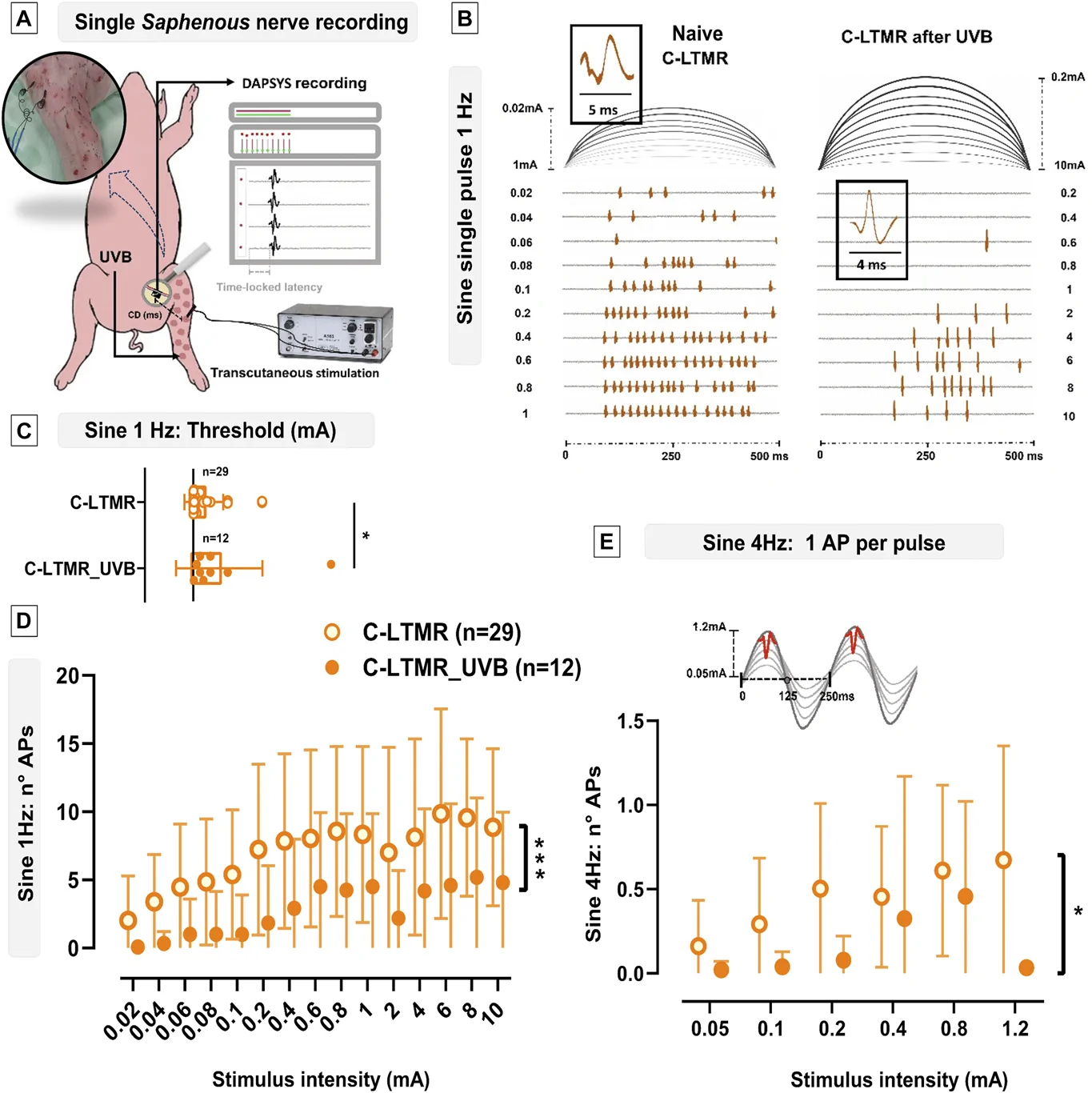
Desensitization of C-LTMR. Single nerve fibres from pig *saphenous* nerve in vivo were recorded using DAPSYS 8.0 after UV-B irradiation (A). The total number of action potentials in response to transcutaneous slow depolarising electrical stimulation was compared among nonirradiated “naive” C-LTMRs recorded previously (left panel) and units recorded 3 to 10 hours after UV-B–induced inflammation (right panel, B). Note the clear increase in the threshold of activation in UV-B–irradiated C-LTMRs (n = 12, Mann–Whitney *U* test, *P < 0.01) (C) as well as a decrease in total number of action potentials (D) in response to single pulse 1-Hz (500 milliseconds) sine wave stimulation of 0.02- to 10-mA amplitudes (Holm–Bonferroni correction, ***P < 0.05). Average number of action potentials per second recorded during sine 4-Hz stimulation of 1-minute duration and 0.05- to 1.2-mA amplitudes (E), applied to the UV-B–irradiated receptive fields of 12 C-LTMR fibres (solid symbols) and nonirradiated (n = 29) C-LTMR units (Holm–Bonferroni correction, *P = 0.02). Data are presented as mean ± SD. C-LTMR, low-threshold mechano-receptor; UV-B, ultraviolet B.

The skin of the saphenous nerve innervation territory was irradiated using a hand-held UV-B lamp (Dermalight 80 MED-Tester; Dr. Höhnle Medizintechnik, Kaufering, Germany). The minimal erythemal dose (MED) for UV-B pig skin irradiation is 0.112 J/cm^2^.^33^ We exposed the pig hind limb to UV-B for 1 minute delivering 0.36 J/cm^2^ to achieve the 3-fold MED. About 10 to 15 skin spots (each 1.5 cm^2^) were irradiated to cover the innervation territory of the saphenous nerve at the pig medial limb (Fig. 1A). Clear erythema scores ≥3 could be visualized after 3 to 4 hours. Single nerve fiber recordings were performed only if the fibers' receptive field was located precisely inside the UV-B erythematous area. Recordings were performed after 3 and up to 10 hours after UV-B irradiation.

### 2.3. Single nerve fiber recordings in vivo

The saphenous nerve was exposed by a skin incision of about 6 cm on the medial hind limb. The subcutaneous connective tissue was removed and the skin flap stitched to a metal ring, forming a pool filled with paraffin oil for electrical insulation. A fascicle of the saphenous nerve was transected and placed on the recording mirror inside the paraffin pool. The teased fiber technique was performed as previously described^4,25,30^ and a fine strand of the saphenous nerve attached to a golden wire for action potential (AP) recording. The receptive field of the fascicle was identified by scratching and squeezing the hind limb skin. Electrical pulses (20 mA/0.25 Hz/1 millisecond, Digitimer DS7A, Welwyn, United Kingdom) were delivered to the receptive field and inside the UV-B–irradiated spot until time-locked APs could be recorded. Action potential signals were amplified (INV Biosignal Amplifier, Avere Solutions UG, Erlangen, Germany), filtered (bandwidth 100–3000 Hz, Model 3364, Krohn-Hite Corp., Brockton, MA), audio monitored, and recorded with DAPSYS 8.0 software package (Brian Turnquist, St. Paul, MN).

### 2.4. Electrical stimulation protocols

Nociceptors were stimulated by high-frequency 100-Hz electrical stimulation using noninsulated microneurography needles inserted intracutaneously into the receptive field of the recorded unit. Suprathreshold electrical pulses of 2 Hz were also applied for 3 minutes to record activity-dependent slowing (ADS) for nociceptor classification^38^ (see section below). Slow depolarizing electrical sine wave stimulation of 1 Hz/500 milliseconds (0.02–10 mA) and 4 Hz/250 milliseconds (0.05–0.8 mA) were applied transcutaneously via a pair of L-shaped blunted bipolar platinum–iridium electrodes (Cephalon, the Netherlands), using a constant current stimulator (A395, WPI, Worcester, MA) commanded from DAPSYS 8.0.

The responses to the single sine wave stimulation at 1 Hz are presented as number of evoked APs. When a unit did not respond to a particular stimulus intensity, a carry backward technique was applied, that is, a response value equal to zero was allocated to all lower stimulation amplitudes, even if these were nontested intensities. It was demonstrated before that C fibers respond to suprathreshold 1-Hz stimuli with a burst of APs.^34^ Activation thresholds of a unit to a given stimulus intensity were defined by a minimum of 3 AP discharges in response to a tested stimulus, followed by a consistent response to sequential higher intensities.

The application of 4-Hz 1-minute stimuli resulted in a total of 240 pulses for each intensity. Responses of the unit are given as number of APs per pulse, and the carry backward method was also applied to the sine wave 4-Hz stimulus.

### 2.5. Thermal stimulation

Heat stimuli from 35 to 55°C generated by a CO_2_-laser (diameter 12 mm, SIFEC, Ferrieres, Belgium) were delivered to the receptive field, and units tested for thermal thresholds (“staircase” paradigm) by temperature increase from 35 to 50°C in steps of 1°C. Each temperature was held for 1 second, and the response of the unit recorded. After the staircase, fibres were challenged with 45 and 55°C stimuli applied for 5 seconds, and the number of APs counted during the stimulation period.

### 2.6. Nerve fiber classification

C-fibres were discriminated from other afferents based on the conduction velocity (CV) of the unit (eg, A-afferents have CVs above 2 m/s^28^). C-fibres were then classified according to their responsiveness to mechanical, heat, and electrical stimulation online and verified offline. Low-threshold mechano-receptors (C-LTMR, “C-touch” afferents) were classified based on their responsiveness to light “brush” (assessed by stroking the receptive field with a soft paintbrush) and an absent response to heat stimulation.^42^ In nonirradiated skin, high-threshold-mechano-thermal-sensitive C-nociceptors (“polymodal” nociceptors, CMH) can be differentiated from very-high-threshold-mechano-C-nociceptors (C-VHT) based on mechanical thresholds, assessed with Semmes–Weinstein monofilaments (North Coast Medical Inc, Morgan Hill, CA) as described previously.^39^ In addition, CMH consistently respond to CO_2_-laser heat stimulation^31^ while C-VHT only occasionally respond to that stimulus. C-VHT nociceptors also differ in their slowing pattern to high-frequency 100-Hz suprathreshold rectangular electrical stimulation.^49^ CMH not only can follow the high-frequency stimulation but also clearly differ in latency slowing from first to second AP at 100 Hz (Fig. S1A, available at https://links.lww.com/PR9/A441) when compared with C-VHT. Mechano-insensitive or silent C fibres were differentiated from C-VHT based on their mechanical irresponsiveness, the complete inability to follow electrical high-frequency 100-Hz stimulation, and their ADS after electrical 2-Hz stimuli delivered for 3 minutes (Fig. S1B, available at https://links.lww.com/PR9/A441).^9,29,38^

The same classification criteria outlined above was applied after UV-B–induced inflammation. However, in sunburn, primary afferent nerve fibres can alter mechanical and/or thermal responsiveness. Therefore, the electrical excitability patterns to high frequent 100-Hz and to 2-Hz 3-minute stimulation were crucial for stringent separation of C-nociceptors. Particularly the 2-Hz stimulation paradigm evokes an ADS exceeding 20% in CMi after UV-B (Fig. S1B, available at https://links.lww.com/PR9/A441)^48^ and thus can be used as classification criterion to separate CMi units from C-VHT nociceptors, which do not alter activity-dependent slowing to 3-minute 2-Hz stimulation in UV-B–irradiated skin (Fig. S1B, lower panel, available at https://links.lww.com/PR9/A441). Some CMi units, however, displayed mechanical or heat responsiveness, but still had an ADS >20% at 2 Hz 3 minutes. These nociceptors, therefore, were classified as “sensitized” CMi and distinguished from “nonsensitized” CMi nociceptors in UV-B skin. The increased number of CMi nociceptors recorded in UV-B–irradiated skin might relate to higher receptive fields when compared with CMH nociceptors.^37^

### 2.7. Data and statistical analyses

Conduction distance (mm) was measured between the recording electrode and the microneurography needles inserted in the receptive field. The AP elicited by a rectangular electrical 20-mA pulse provided a fixed-latency measured in milliseconds. Conduction velocity (m/s) was calculated dividing conduction distance by the latency upon rectangular stimulation. The number of APs to both slow depolarising stimuli and laser were analysed with DAPSYS 8.0 software (Brian Turnquist, St. Paul, MN). Graphical representation and statistical analyses were performed in GraphPad Prism version 8.0.2 (GraphPad Software, Boston, MA) and Statistica version 7.1 (Statsoft, Tulsa, OK). Chi-square and Fisher exact test were used when comparing percentage of responders with a certain stimulus. Nonparametric Mann–Whitney *U* tests with Holm–Bonferroni correction for multiple testing were used to compare different stimulus intensities and numbers of APs for sine wave 1 and 4-Hz stimulation before and after UV-B irradiation for each nerve fiber class. The Robust regression and outlier removal with Q-parameter of 1% was used to exclude outliers. Grouped data are presented as mean ± SD, with group size n, rank biserial effect size *r*, 95% confidence intervals (CIs), and Mann–Whitney *U* test level of significance *P* < 0.05.

## 3. Results

Single fibers were isolated online according to a time-locked latency and recorded 3 to 10 hours after UV-B–induced inflammation (Fig. 1A). The UV-B–irradiated units were classified, based on the criteria described above and previously,^30^ into 12 C-LTMR, 9 CMH, 11 VHT, and 13 CMi. A total of 29 C-LTMR tactile fibers, 57 CMH, 30 VHT, and 18 CMi nociceptors were used as naïve nonirradiated control. Vital parameters (EtCO_2_ [40.7% ± 3.5%], SPO_2_ [99.4% ± 1.1%], body core temperature [36.9 ± 0.6°C], heart rate [109 ± 13.6 bpm], or rate of intravenous Narcoren infusion [17.29 ± 3.2 mg/kg/h]) did not correlate with C-nociceptor CV (naïve skin 1.01 ± 0.39 m/s vs UV-B 1.1 ± 0.61 m/s), excitation thresholds to 1-Hz stimulation in naïve (all parameters *r* ≈ 0.01, *P* > 0.27) and UV-B–irradiated skin (all parameters *r* ≈ −0.06, *P* > 0.22), or number of APs to 1-minute 4-Hz stimulation (naïve skin *r* ≈ −0.05, *P* > 0.36 and UV-B–irradiated skin *r* ≈ 0.17, *P* > 0.34). Notably, the recording time post UV-B irradiation was grouped in 2 slots (3–5 hours vs 6–10 hours after UV-B), considering that time post UV-B may affect mediator dynamics, impact transcription, translation, axonal transport, and channel trafficking. Covariate analysis revealed no significant effect of “time post UV-B” (3–5 hours vs 6–10 hours) on stimulus intensity evoked numbers of APs (*F*(1, 39) = 0.92, *P* = 0.976, effect size ηp^2^ < 0.001) for the fiber classes and their interaction (*F*(36, 351) = 0.99, *P* = 0.494, ηp^2^ = 0.09).

Unexpectedly, a clear desensitization of tactile C fibers was observed after UV-B irradiation, with a reduction in electrically induced activation (Fig. 1B) and a 3-fold increase in electrical threshold for sinusoidal 1-Hz stimulation (Mann–Whitney *U* test, *P* = 0.01, *r* = 0.4) (Fig. 1C). The threshold of activation for naïve C-LTMR fibers (n = 29) upon sine 1 Hz was 0.6 ± 1.12 mA (95% CI = 0.41) and increased to 1.52 ± 0.83 mA (95% CI = 2.45) after UV-B (n = 12). After UV-B irradiation, there was a significant stimulus intensity–dependent reduction in the number of APs to sinusoidal 1 Hz (Holm–Bonferroni correction, *P* < 0.05, *r* = 0.26–0.44) (Fig. 1D) and to sine 4 Hz 1 minute (Holm–Bonferroni correction *P* < 0.02, *r* = 0.45–0.49) (Fig. 1E). Moreover, there was also a significant decrease in number of after discharges to sine 1-Hz stimuli in C-LTMR of UV-B–irradiated skin (Mann–Whitney, *P* < 0.0002, *r* = 0.58) (Fig. S2A, available at https://links.lww.com/PR9/A441).

**Figure 2.**
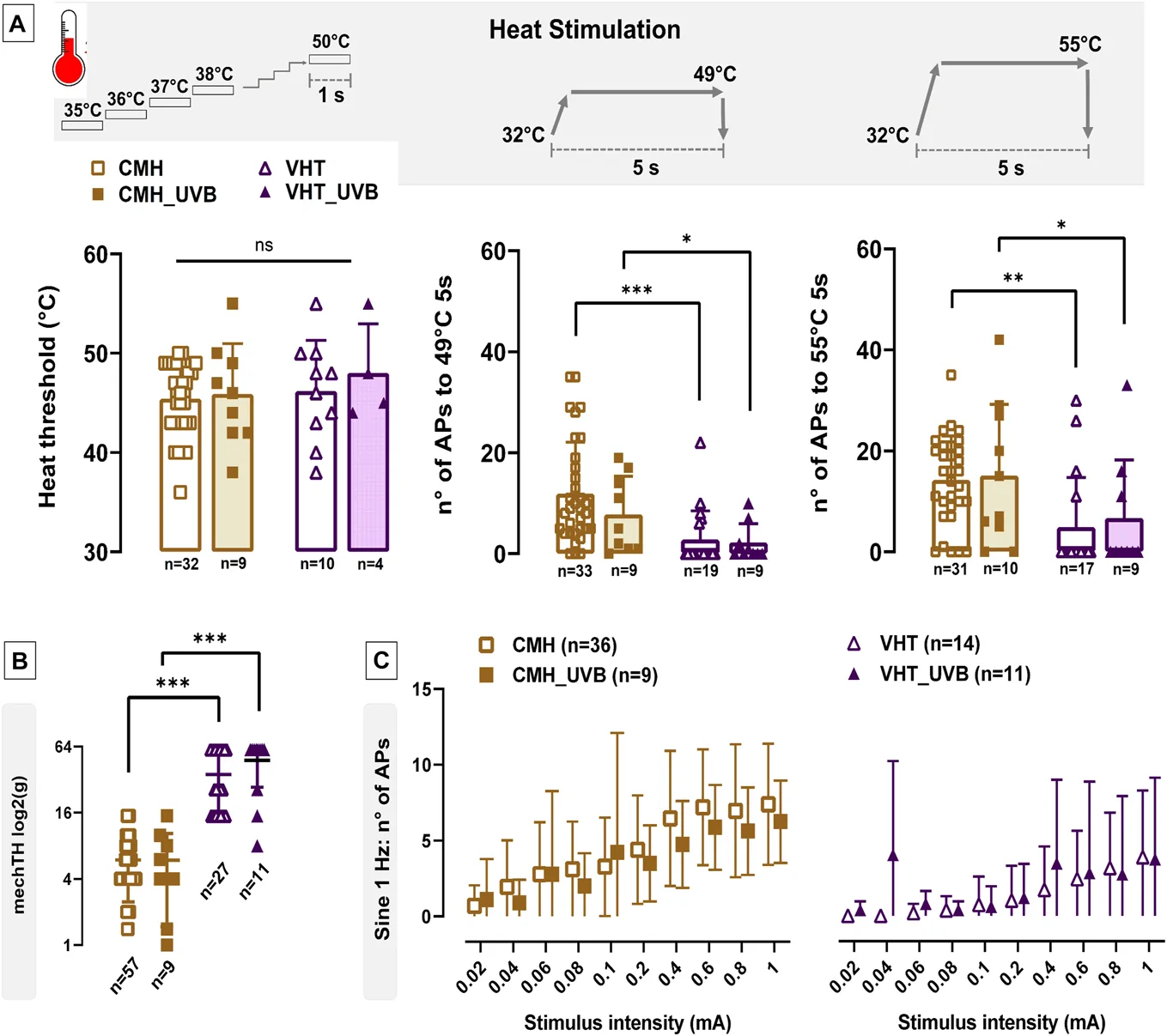
Polymodal nociceptors lack sensitization after UV-B. (A) The heat threshold tested with CO_2_-laser in 1°C staircases from 35°C to 55°C (each temperature 1-second duration) was unaffected (Mann–Whitney *U* test, n.s.) after UV-B–induced inflammation in both CMH (n = 9) and C-VHT (n = 9) nociceptors (left panel). There was also no increase in the total number of action potentials (APs) in naïve compared with UV-B–irradiated skin in response to 49°C (Mann–Whitney *U* test, *P* > 0.3) and 55°C (Mann–Whitney *U* test, *P* > 0.8) delivered for 5 seconds to the receptive fields of CMH and VHT units (right panel). Note that heat responses in VHT compared with CMH were lower in both naïve and UV-B skin at 49°C and 55°C (Mann–Whitney *U* test *P* < 0.02 and *P* < 0.05). (B) No difference in mechanical threshold tested with Semmes–Weinstein monofilaments was observed after UV-B. (C) There was no significant effect on the total number of action potentials in CMH (left panel) or VHT nociceptors (right panel) evoked by 1-Hz sine wave stimulation (Mann–Whitney *U* test, n.s.). Data are presented as mean ± SD. UV-B, ultraviolet B; VHT, very-high-threshold.

Based on the UV-B heat hyperalgesia reported in humans, we expected also sensitization to heat in pig nociceptors after UV-B–induced inflammation. However, we did not observe any significant changes in thermal thresholds (Fig. 2A) for CMH (Mann–Whitney, *P* > 0.7, *r* = 0.057) or VHT-fibers (Mann–Whitney, *P* > 0.8, *r* = 0.047). There was no change in the number of APs in response to 5 seconds 49°C (Fig. 2A, middle) or 55°C (Fig. 2A, right) heat stimulation before and after UV-B irradiation for both CMH (n = 9) and VHT (n = 9) nociceptors (Mann–Whitney, CMH *P* > 0.3, *r* = 0.16 [49°C]; *P* > 0.8, *r* = 0.036 [55°C] and VHT *P* > 0.49, *r* = 0.17 [49°C]; *P* > 0.9, *r* = 0.037 [55°C]). Foreseeably, heat responses were lower in VHT as compared to CMH at 49°C (Mann–Whitney, naïve *P* < 0.0005, *r* = 0.53, UV-B *P* < 0.02, *r* = 0.62) and 55°C (Mann–Whitney, naïve *P* < 0.01, *r* = 0.39, UV-B *P* < 0.04, *r* = 0.51). Mechanical thresholds of CMH were lower than those of VHT (Fig. 2B, Mann–Whitney, naïve *P* < 0.0001, *r* = 0.73, UV-B *P* < 0.0001, *r* = 0.83), but there was no significant effect of UV-B inflammation in both nociceptor subclasses (Mann–Whitney, CMH *P* > 0.5, *r* = 0.087 and VHT *P* > 0.2, *r* = 0.27).

Activation thresholds of CMH units to 1-Hz stimulation increased 2-fold in naïve skin (0.09 ± 0.08 mA) compared with UV-B (0.2 ± 0.2 mA), although not significantly (Mann–Whitney, *P* > 0.44, *r* = 0.13). There was no significant effect of UV-B irradiation on the total number of APs in response to sine 1 Hz in CMH (Fig. 2C, left) (Mann–Whitney, *P* > 0.7, *r* = 0.05) or VHT (Fig. 2C, right) nociceptors (Mann–Whitney, *P* > 0.8, *r* = 0.05). The number of APs recorded up to 1-mA stimuli was in CMH nociceptors on average 39 ± 25 (95% CI = 8.2) in naïve (n = 36) and 34 ± 16 (95% CI = 10.5) in UV-B skin (n = 9). In VHT nociceptors, we recorded on average a total of 40 ± 32 APs (95% CI = 17.4) in naïve (n = 14) and 33 ± 19 APs (95% CI = 12.1) in UV-B skin (n = 11). Accordingly, Holm–Bonferroni correction for multiple current intensity testing at 1 Hz revealed no statistical significance of UV-B compared with naïve skin for either CMH (n.s., *r* = 0.11–0.17) or VHT nociceptors (n.s., *r* = 0.25–0.39). In contrast, we observed a significant reduction in AP after discharges to sine 1 Hz after UV-B for polymodal CMH nociceptors (Mann–Whitney, *P* < 0.01, *r* = 0.37), with 22 ± 20 APs (95% CI = 6.59) in naïve (n = 36) and 7 ± 10 APs (95% CI = 6.65) in UV-B skin (n = 9) (Fig. S2A, available at https://links.lww.com/PR9/A441). Naïve VHT nociceptors mainly respond to higher stimulus intensity above 2 mA (9 ± 6 APs [95% CI = 4.8], n = 14), but even at this high stimulus-intensity range, there was no increase in nociceptor activity compared with UV-B skin (5 ± 5 APs [95% CI = 3.1], n = 11, Mann–Whitney, *P* > 0.2, *r* = 0.24) (Fig. S2B, available at https://links.lww.com/PR9/A441). Regarding the number of APs in response to sine 4-Hz 1-minute stimulation, there was also no difference before (n = 32) and after UV-B–induced inflammation (n = 9) in CMH fibers (Mann–Whitney, *P* > 0.3, *r* = 0.14) (Fig. S2D, available at https://links.lww.com/PR9/A441). Similarly, when comparing naïve (n = 9) and UV-B–irradiated VHT nociceptors (n = 11), no significant difference was recorded to sine 4-Hz 1-minute evoked APs (Mann–Whitney, *P* > 0.05, *r* = 0.45) (Fig. S2E, available at https://links.lww.com/PR9/A441).

**Figure 3.**
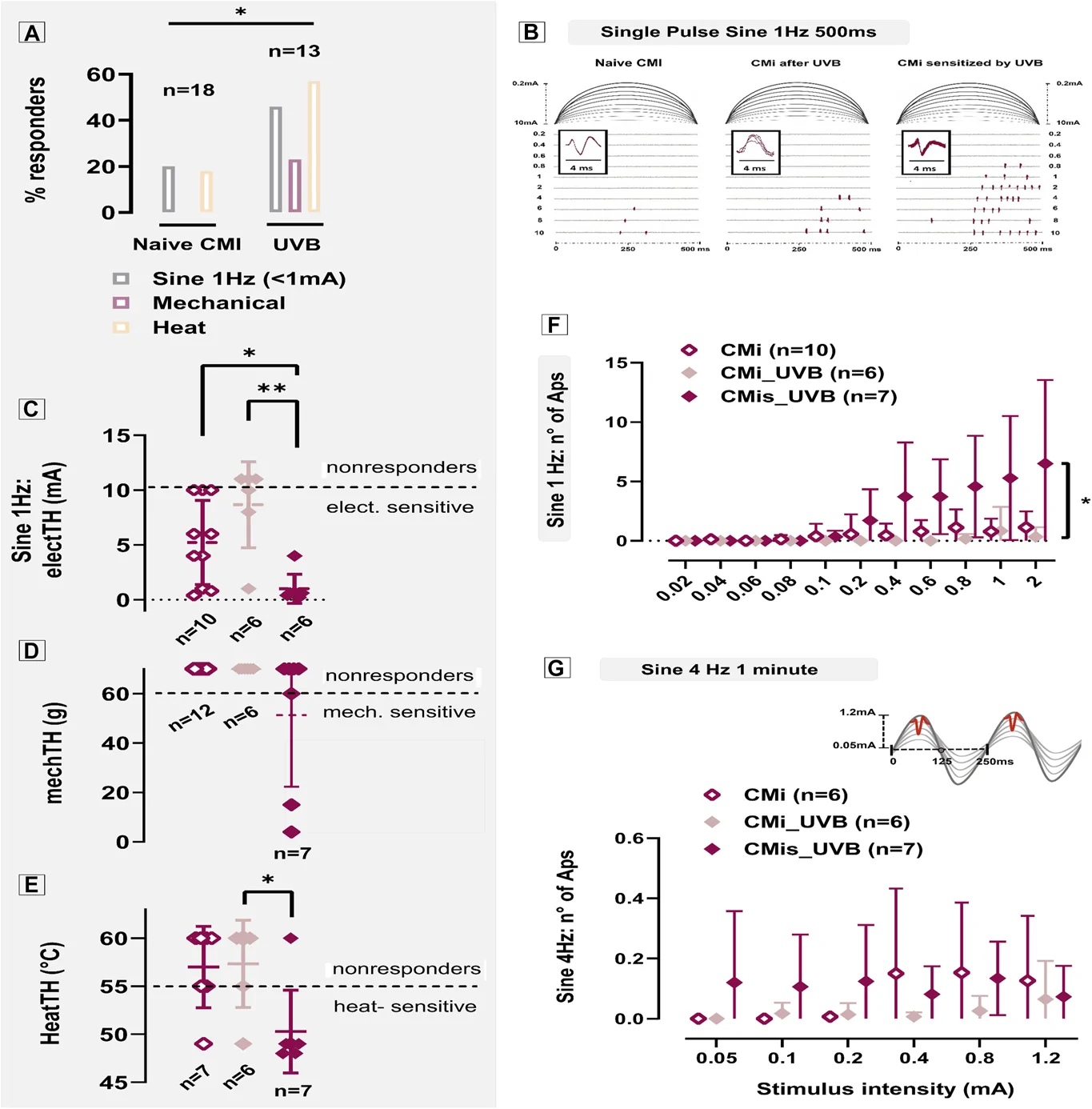
UV-B–induced sensitization in silent nociceptors. (A) The numbers of CMi fibers that responded to heat, mechanical, and sine 1-Hz stimulation were increased (fibers in %) after UV-B–induced inflammation compared with nonirradiated (naïve control) skin. CMi fibers in nonirradiated skin (B, left) or CMi without sensitization after UV-B irradiation (B, middle) responded to sine 1 Hz (500-millisecond duration) only at high stimulus intensities and with very small numbers of action potentials compared with “sensitized” CMi units (B, right). (C) In UV-B sensitized CMi, there is a reduction in threshold of 1-Hz sine wave activation compared with naïve and nonsensitized CMi (Mann–Whitney *U* test, *P < 0.01, **P < 0.005). (D) CMi units show a reduction in mechanical thresholds, although not significantly (n = 3, Mann–Whitney *U* test, *P* > 0.08, *r* = 0.6). (E) Thermal thresholds were significantly lower in “sensitized” compared with nonsensitized CMi (n = 6, Mann–Whitney *U* test, *P < 0.01, *r* = 0.74). (F) There was also an increase in the number of action potentials(APs) in response to sine 1-Hz 500-millisecond duration (Mann–Whitney *U* test, *P* < 0.005, *r* = 0.76). (G) Responsiveness to sine wave 4-Hz 1-minute stimulation was not significantly different among the sensitized silent nociceptors compared with nonsensitized UV-B or naïve CMi nociceptors (Mann–Whitney *U* test, *P* > 0.17 and *P* > 0.3). Data are presented as mean ± SD. UV-B, ultraviolet B.

In UV-B–irradiated skin, we identified an increasing percentage of silent nociceptors that displayed lower thresholds and thus enhanced responsiveness to heat, to sine 1-Hz stimulation, and even to mechanical stimulation (n = 13, χ^2^ test, *P* = 0.012) when compared with naïve CMi units (n = 18, Fig. 3A). When comparing the percentage of silent units with increased responsiveness to other UV-B–irradiated CMi units, the difference among the 2 groups turned to be nonsignificant for sine 1 Hz, heat and mechanical stimulation (Fisher exact test, *P* > 0.05). Although, these units were classified as “sensitized” CMi nociceptors and could be distinguished based on their activity-dependent slowing during 2 Hz (>20%), their absent 100-Hz following-frequency activation, and their mechanical/heat responsiveness, from “nonsensitized” CMi units (Fig. 3B), VHT, and CMH nociceptors in UV-B–irradiated skin. We observed that, among the sensitized CMi fibres (n = 6), thresholds to sine 1-Hz stimulation (Fig. 3C) were reduced 5-fold in comparison to naïve CMi afferents (n = 10, Mann–Whitney, *P* < 0.01, *r* = 0.63) and UV-B–irradiated nonsensitized CMi units (n = 6, Mann–Whitney, *P* < 0.005, *r* = 0.79). The current threshold for activation to 1-Hz stimuli was 5.2 ± 3.85 mA (95% CI = 2.39) in naïve CMi, 8.12 ± 4.22 mA (95% CI = 3.15) for nonsensitized CMi, and 0.9 ± 1.26 mA (95% CI = 0.99) for sensitized silent afferents. In addition, we observed among sensitized CMi fibers (n = 7), few nociceptors (n = 3) with reduced activation thresholds to mechanical stimulation (26 ± 30 g [95% CI = 33.5], Fig. 3D), although not significantly compared with nonsensitized CMi units (n = 6, Mann–Whitney, *P* = 0.09, *r* = 0.6). Heat activation thresholds in sensitized CMi fibers (n = 7) were on average 48.7 ± 0.5°C (95% CI = 0.41) and significantly lower compared with 57 ± 4.5°C (95% CI = 3.6) in nonsensitized nociceptors (n = 6, Mann–Whitney, *P* < 0.01, *r* = 0.74, Fig. 3E). The total number of APs in response to sine 1 Hz was higher in sensitized CMi fibers (n = 7, 25 ± 17.5 (95% CI = 13.9)) compared with nonsensitized CMi (n = 6, 6 ± 2.5 [95% CI = 1.95], Mann–Whitney, *P* < 0.005, *r* = 0.76) or naïve CMi nociceptors (n = 10, 5.6 ± 6.5 [95% CI = 4.02], Mann–Whitney, *P* < 0.05, *r* = 0.49) (Fig. 3F). We hypothesized also a current stimulus dependent increase in number of APs in “sensitized” CMi fibers. Despite effect sizes of *r* > 0.6 for 0.6- and 1-mA stimuli, however, pairwise comparisons across the delivered stimulus intensities yielded no difference between the classified CMi nociceptors (all values *P* > 0.05, Holm–Bonferroni correction). The number of AP after discharges were also not significantly different between the CMi nociceptor groups (Mann–Whitney, *P* > 0.2, *r* = 0.33, Fig. S2A, available at https://links.lww.com/PR9/A441). Similar to VHT nociceptors, naïve silent nociceptors tended to respond to sine 1 Hz only at high stimulus intensities. When looking at the intensity range of 4 to 10 mA, there was no increase in the response of CMi nociceptors (Fig. S2C, available at https://links.lww.com/PR9/A441). “Nonsensitized” CMi fibers in UV-B skin (n = 6) responded with fewer APs to 1-Hz stimuli compared with nonirradiated “naïve” silent afferents (n = 6), however, not significantly (Holm–Bonferroni correction n.s., *r* = 0.18–0.63). There was also no significant difference in the number of APs in response to sine 4-Hz 1-minute stimulation (Fig. 3G) in naïve silent nociceptors (n = 6) compared with nonsensitized CMi (n = 6, Mann–Whitney, *P* > 0.3, *r* = 0.33) or sensitized CMi (n = 7, Mann–Whitney, *P* > 0.3, *r* = 0.27). Although there was a tendency of sensitized CMi to start firing at lower stimulus intensities, the total number of APs among the 1-minute 4-Hz stimuli was not significantly different compared with nonsensitized CMi in UV-B skin (Mann–Whitney, *P* > 0.17, *r* = 0.41) or naïve CMi nociceptors (Mann–Whitney, *P* > 0.36, *r* = 0.28, Fig. 3G).

## 4. Discussion

Unexpectedly, UV-B did not sensitize polymodal nociceptors to mechanical or thermal stimuli. If anything, they were desensitized to slow depolarizing pulses, which might mask the inflammatory sensitization of their transduction proteins. An even more pronounced desensitization to slow depolarizing pulses was found in C-LTMR fibers. Only silent nociceptors were clearly sensitized, not only to mechanical and thermal stimuli, as shown before, but also to slow depolarizing electrical stimuli, suggesting a major role of this nociceptor class in inflammatory hyperalgesia. This finding adds on to our previous observation of silent nociceptors being sensitized to follow high-frequent electrical stimuli in a sunburn.^48^ Moreover, our data indicate differential effects within CMi nociceptors, perhaps related to complex time-dependent changes under inflammatory conditions.

We speculate that depolarised inactivated voltage-gated sodium channels accentuating desensitization in some but not all nociceptors under inflammatory conditions might explain polymodal nociceptor and C-LTMR fiber desensitization. In addition, C-LTMR fibers are associated to affective touch in humans^24^ but were also reported to inhibit pain processing at spinal and cortical level,^13,20^ and thus, their inhibition might add to UV-B–induced hyperalgesia processing. Also, temporal mediator dynamics, transcription, translation, and axonal channel trafficking may affect extrinsic nociceptor activity and facilitate intrinsic after discharge development to overlap with spontaneous activity (for review, see Walters et al.^44^).

Pig skin architecture was previously described as a translational model to human skin^23,41,45^ in which the UV-B–induced inflammation was extensively explored.^15,32,47,48^ Slow depolarising stimuli have proven to be a reliable method to preferentially stimulate specific C-fibre afferents in mouse, humans, and pigs.^6,16,17,34^ Here, we recorded pig sensory afferents between 3 and 10 hours after UV-B irradiation. Mechanical and thermal hyperalgesia under inflammatory conditions is thought to be partly mediated by hyperexcitability of polymodal nociceptors,^11,36^ corresponding to CMH and VHT in our study. Interestingly, UV-B–induced sensitization of polymodal C-nociceptors has only been shown for suprathreshold heat in rabbit and rat^3,39^ and for suprathreshold mechanical stimuli in heat-insensitive CM nociceptors^3^ in rat. In contrast, mechanical responses of A-delta nociceptors and polymodal C-nociceptors were desensitized by UV-B in rats.^3^ The few A-delta fibers (n = 5) recorded here support lack of UV-B–induced sensitization also to electrical sine 1-Hz (Fig. S1C, available at https://links.lww.com/PR9/A441) and 4-Hz stimulation (Fig. S1D, available at https://links.lww.com/PR9/A441). Alternatively, potential effects of UV-B irradiation on Aβ fibers might also be assumed, but appear unlikely as corpuscles innervated by Aβ fibers are located in deeper skin layers and should not be reached by the UV-B light. Thus, previous results support the lack of sensitization of polymodal or VHT nociceptors in the UV-B model. Heat transduction is expected to be facilitated, based on a sensitization of TRPV1 by inflammatory mediators,^10,26,43^ but also by a shift of the spike initiation site closer to the sensory endings.^12^ It could be argued that increased epidermal thickening after UV-B exposure may thwart superficial heat conduction, but also the observed axonal hypoexcitability that emerged as desensitization to electrical slow depolarizing stimuli in our study might have masked the sensitization of heat transduction on sensory endings. Therefore, potential sensitization of heat transduction will not be detected if axonal excitability of heat sensitive CMH nociceptors is reduced. We cannot completely rule out, but find unlikely based on previous findings^48^ and current results, that CMH nociceptors had gained ADS and were, therefore, misclassified into CMi nociceptors.

Our results indicate instead silent nociceptor sensitization, with increased responses to mechanical, thermal, and particularly electrical sinusoidal stimuli. Mechanical hypersensitivity of silent nociceptors after inflammation is extensively debated and lately associated with overexpressed transmembrane protein TMEM100.^27^ Thermal and electrical hyperexcitability in silent fibres after UV-B was reported before in pig skin,^48^ supporting our results.

Nociceptor sensitization after UV-B irradiation has been reported to be restricted to the irradiated skin^3^ suggesting a dominant role of local mechanisms. The release of sensitizing inflammatory mediators^1^ is known to depolarize nociceptors via several mechanisms including activation of TRPV1, TRPA1, and anoctamin 1 but also via inhibition of potassium leak currents.^5^ The latter may contribute to an axonal sensitization. Additional contributors to UV-B–induced axonal hyperexcitability include increased trafficking and upregulation of Na_V_1.7 at the membrane of distal axons^14^ and involve also an increased local PGE_2_ release sensitizing tetrodotoxin-resistant sodium channel Na_V_1.8 via phosphorylation.^8^ Sensitization of nociceptors by depolarization is expected to be particularly effective for those cells that mainly depend on Na_V_1.8 activation,^46^ including silent nociceptors in particular.^18^ PGE2 has been shown to induce sustained axonal membrane potential depolarization in vitro, and these depolarizations were primarily dependent on Na_V_1.8 conductivity.^21^ Silent nociceptors in pig appear to have a high Na_V_1.8 expression,^25,30^ whereas polymodal nociceptors and C-LTMRs were found to be mainly tetrodotoxin-sensitive.^18^ Thus, we assume that a differential expression of Na_V_1.8 on nociceptors could explain the divergent sensitization pattern observed between the C-fiber classes in UV-B–irradiated skin. Recent successful trials of Na_V_1.8 blockers in postoperative pain patients^19^ support a clinically important role of this sodium channel in inflammatory pain conditions.

## 5. Limitations

A previous study found an increase in spontaneous activity of C-nociceptors that we did not observe,^48^ which is potentially based on the earlier interval after UV-B irradiation. Even if we did not notice a significant effect of the time post UV-B irradiation (3–5 hours vs 6–10 hours) on the stimulus evoked AP number in all recorded fiber classes, we cannot exclude spontaneous depolarizations occurring at later time points such as 24 hours after irradiation, as observed before in humans.^32^ We did not test the effect of suprathreshold mechanical stimuli and thus mechanical sensitization to such stimuli cannot be excluded. A fundamental difficulty in our study was the classification of nociceptors based on responses to mechanical and heat stimuli, which are expected to change after UV-B irradiation. Our classification was, therefore, mainly based on axonal properties that have been shown to retain the differences between the fiber classes.^48^ Finally, mechanistic considerations on Na_V_1.8 remain speculative as we did not include pharmacological testing.

In summary, our data show a divergent effect of UV-B irradiation, that is, sensitizing silent nociceptors and desensitizing polymodal nociceptors and C-LTMR units, which emphasizes the different roles of specific nociceptor classes for acute versus inflammatory pain. They also support the urgency of current translational attempts to classify primary afferent neurons based on expression pattern^40^ in combination with single neuron patch-seq approaches^23^ and functional characteristics derived from pluripotent stem cells^7^ that can be tested similarly in humans.

## Disclosures

The authors have no conflicts of interest to declare.

## Supplemental digital content

Supplemental digital content associated with this article can be found online at https://links.lww.com/PR9/A441.
